# Multiparameter Evaluation of the Platelet-Inhibitory Effects of Tyrosine Kinase Inhibitors Used for Cancer Treatment

**DOI:** 10.3390/ijms222011199

**Published:** 2021-10-18

**Authors:** Bibian M. E. Tullemans, Alicia Veninga, Delia I. Fernandez, Maureen J. B. Aarts, Johannes A. Eble, Paola E. J. van der Meijden, Johan W. M. Heemskerk, Marijke J. E. Kuijpers

**Affiliations:** 1 Department of Biochemistry, Cardiovascular Research Institute Maastricht (CARIM), Maastricht University, 6229 ER Maastricht, The Netherlands; bibian.tullemans@maastrichtuniversity.nl (B.M.E.T.); a.veninga@maastrichtuniversity.nl (A.V.); d.fernandezdelafuent@maastrichtuniversity.nl (D.I.F.); p.vandermeijden@maastrichtuniversity.nl (P.E.J.v.d.M.); 2 Platelet Proteomics Group, Centre for Research in Molecular Medicine and Chronic Diseases (CIMUS), Universidad de Santiago de Compostela, Avda de Barcelona s/n, 15782 Santiago de Compostela, Spain; 3 Department of Medical Oncology, GROW School for Oncology and Developmental Biology, Maastricht University Medical Centre, P. Debyelaan 25, 6229 HX Maastricht, The Netherlands; mjb.essers.aarts@mumc.nl; 4 Institute of Physiological Chemistry and Pathobiochemistry, University of Münster, Waldeyerstraße 15, 48149 Münster, Germany; johannes.eble@uni-muenster.de; 5 Thrombosis Expertise Center, Maastricht University Medical Center+ (MUMC+), 6229 HX Maastricht, The Netherlands; 6 Synapse Research Institute, Kon. Emmaplein 7, 6214 AC Maastricht, The Netherlands; jwmheem722@outlook.com

**Keywords:** platelet activation, aggregation, bleeding, thrombus formation, tyrosine kinase inhibitors, antiplatelet treatment

## Abstract

Current antiplatelet drugs for the treatment of arterial thrombosis often coincide with increased bleeding risk. Several tyrosine kinase inhibitors (TKIs) for cancer treatment inhibit platelet function, with minor reported bleeding symptoms. The aim of this study was to compare the antiplatelet properties of eight TKIs to explore their possible repurposing as antiplatelet drugs. Samples of whole blood, platelet-rich plasma (PRP), or isolated platelets from healthy donors were treated with TKI or the vehicle. Measurements of platelet aggregation, activation, intracellular calcium mobilization, and whole-blood thrombus formation under flow were performed. Dasatinib and sunitinib dose-dependently reduced collagen-induced aggregation in PRP and washed platelets; pazopanib, cabozantinib, and vatalanib inhibited this response in washed platelets only; and fostamatinib, axitinib, and lapatinib showed no/limited effects. Fostamatinib reduced thrombus formation by approximately 50% on collagen and other substrates. Pazopanib, sunitinib, dasatinib, axitinib, and vatalanib mildly reduced thrombus formation on collagen by 10–50%. Intracellular calcium responses in isolated platelets were inhibited by dasatinib (>90%), fostamatinib (57%), sunitinib (77%), and pazopanib (82%). Upon glycoprotein-VI receptor stimulation, fostamatinib, cabozantinib, and vatalanib decreased highly activated platelet populations by approximately 15%, while increasing resting populations by 39%. In conclusion, the TKIs with the highest affinities for platelet-expressed molecular targets most strongly inhibited platelet functions. Dasatinib, fostamatinib, sunitinib, and pazopanib interfered in early collagen receptor-induced molecular-signaling compared with cabozantinib and vatalanib. Fostamatinib, sunitinib, pazopanib, and vatalanib may be promising for future evaluation as antiplatelet drugs.

## 1. Introduction

Ischemic heart disease and stroke accounted for >15 million deaths worldwide in 2019, making arterial thrombosis a leading global cause of death [1]. The major underlying causes are the thrombotic complications of atherosclerosis either via plaque rupture or erosion [2,3]. Current treatment is mostly (dual) antiplatelet therapy, such as aspirin and clopidogrel, which prevent thromboxane A_2_ release and ADP receptor activation, respectively [4]. However, these inhibitors mostly have an irreversible mode of action on the platelet G-protein coupled receptor (GPCRs)-signaling pathways and coincide with an increased risk of bleeding [5]. Therefore, novel antiplatelet drugs are being developed that inhibit thrombosis but do not interfere in hemostasis [5]. In this respect, glycoprotein (GP) VI and C-type lectin-like receptor 2 (CLEC-2) receptors, as well as downstream tyrosine kinases, have been postulated as promising antithrombotic targets [6].

GPVI is an immunoreceptor tyrosine-based activation motif (ITAM)-linked receptor, activated by collagen and fibrin(ogen), resulting in initial platelet activation and subsequent thrombus formation [7,8,9]. A deficiency of GPVI in mice suppressed arterial thrombosis without substantial prolongation of bleeding time [10,11]. Inhibition of GPVI has also been reported to provide protection against thrombosis upon plaque rupture in mice [12,13]. Furthermore, patients with GPVI defects have only a mild bleeding diathesis [14] or remain asymptomatic [15]. Two GPVI inhibitors showing promising antithrombotic effects are currently being tested in Phase II clinical trials: the Fab fragment ACT017 and Revacept, which is a collagen-binding dimeric GPVI-Fc fusion protein [6]. However, a disadvantage of these treatments is their administration via intravenous injections. 

CLEC-2 is a hemITAM-linked receptor activated by the endogenous ligand podoplanin [16]. CLEC-2-deficient mice were protected from arterial thrombus formation and displayed only a minor effect on hemostasis [17,18]. In contrast, a deficiency of both GPVI and CLEC-2 resulted in a lack of cessation of tail bleeding as well as a profound reduction in arterial thrombosis [19]. In human blood, a monoclonal antibody (mAb) to CLEC-2 has been shown to block CLEC-2-induced platelet activation in vitro [20]. However, no clinical trials targeting CLEC-2 have started until now. 

Both GPVI and CLEC-2 heavily depend on tyrosine kinase-signaling, including Syk, Btk, and Src family kinases (SFK) [21]. In addition, other receptor-signaling pathways, likely to a lesser extent, include tyrosine kinases, such as those evoked by GPIb, integrin αIIbβ3, Axl, and Tie, which are receptors for vWF, fibrinogen, Gas6, and angiopoietin, respectively [22,23]. With such a wide mode of action on platelet activation, while leaving GPCR-signaling mostly intact, tyrosine kinase inhibitors (TKIs) may provide an alternative way to inhibit platelet activation [6]. During the past decades, many orally available TKIs have been developed for cancer treatment [24,25], with different inhibitory profiles against tyrosine kinases (Table 1). These compounds have been shown to be well tolerated and, in general, cause only a mild increase in the risk of bleeding. The Syk inhibitor fostamatinib, used for the treatment of chronic immune thrombocytopenic purpura (ITP), has been reported to reduce GPVI and CLEC-2-stimulated platelet aggregation, with only minor effects on bleeding time in mice [26,27]. Notably, TKIs targeting a broad spectrum of tyrosine kinases, including the receptors for the vascular endothelial growth factor (VEGFR), platelet-derived growth factor (PDGFR), and Kit, are used for the treatment of renal cell carcinoma. These include sunitinib, pazopanib, axitinib, and cabozantinib (Table 1). We have recently shown that sunitinib and pazopanib inhibited collagen-induced platelet function both in vitro and in patients receiving treatment [28,29,30]. Axitinib and cabozantinib also have targets that are present in platelets [25] and have been associated with an increased risk of bleeding [31], but no effects on platelet function have been reported. On the contrary, several TKIs used for treating breast and colon cancer have limited affinity for the tyrosine kinases present in platelets, without reported bleeding [24]. These include lapatinib and vatalanib (Table 1). 

The aim of the present study was to systematically evaluate the antiplatelet properties of eight clinically used TKIs and to assess their potential for the repurposing of these compounds as antiplatelet drugs. Therefore, we investigated the effects of these compounds on platelet function in isolated platelets and in whole-blood under flow using different agonists to include multiple signaling pathways.

## 2. Results

### 2.1. Characteristics of Selected TKIs

**Figure 1:**
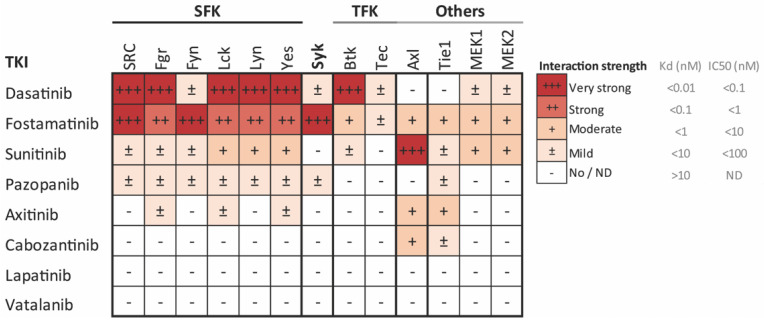
Affinity profile of common tyrosine kinase inhibitors (TKIs) for the relevant tyrosine kinases implicated in platelet activation, presented as a heatmap of the affinity-based dissociation constants (Kd) [39,40,41] or IC_50_ values [42] of the indicated TKIs. Values are from binding experiments with the indicated purified kinases, including Src family kinases (SFK), Syk, Tec family kinases (TFK), focal adhesion kinases (FAK), and MEK. Color coding shows the highest affinity (lowest Kd or IC_50_) in deep red and the lowest affinity (highest Kd or IC_50_) in white. Data are based in [24].

For this study, eight reversible multi-target TKIs were selected on the basis of having published affinities for tyrosine kinases (TKs) expressed in platelets and a minor bleeding risk reported (Table 1, Figure 1). The molecular structures of these TKIs are presented in Figure S1. Dasatinib and fostamatinib displayed high affinity for platelet-expressed Syk and Src-family kinases (SFKs), which mainly signal under GPVI, CLEC-2, and αIIbβ3, while sunitinib, pazopanib, axitinib, and cabozantinib showed moderate to mild affinity for only some of these platelet-expressed targets (Figure 1). The effects of dasatinib and fostamatinib in platelets were not systematically evaluated. Lapatinib and vatalanib had unknown affinities for platelet-expressed kinases (Figure 1, Table 1).

### 2.2. Distinct Effects of Selected TKIs on Collagen-Induced Platelet Aggregation in the Presence or Absence of Plasma

**Figure 2:**
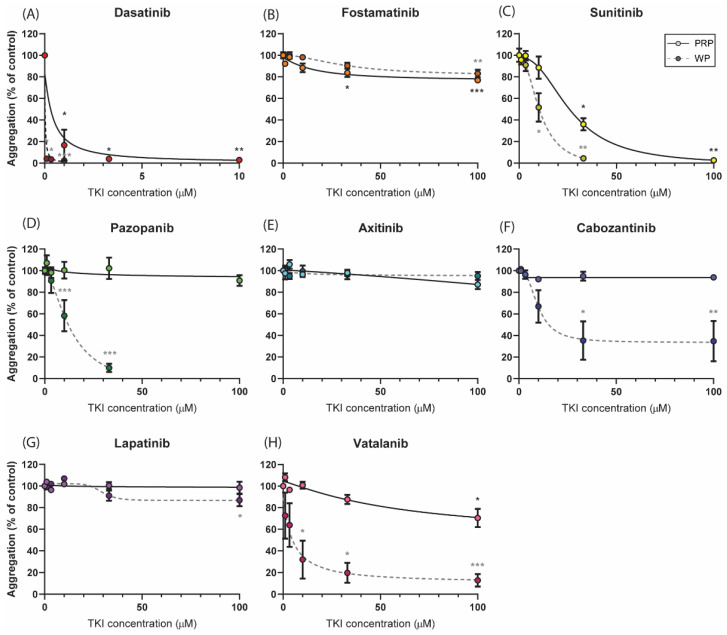
Dose-dependent effects of TKIs on platelet aggregation induced by collagen. Washed platelets or PRP (250 × 10^9^/L) were incubated with the vehicle (<0.1% DMSO) or TKI for 10 min. Dose–response curves of the inhibition of aggregation induced by collagen Type I (1 µg/mL) are shown for (**A**) dasatinib, (**B**) fostamatinib, (**C**) sunitinib, (**D**) pazopanib, (**E**) axitinib, (**F**) cabozantinib, (**G**) lapatinib, or (**H**) vatalanib, measured in either PRP (solid line) or washed platelets (dotted line). Maximum aggregation responses were determined at 8 min; data are shown as means ± SEM (*n* = 6). * *p* < 0.05, ** *p* < 0.01, and *** *p* < 0.001.

As impaired platelet function by TKIs is mostly due to the inhibition of the ITAM-linked receptor, GPVI in particular, we first investigated the effects of the active metabolites of different TKIs on collagen-induced aggregation responses. Dose–response curves with isolated platelets showed the distinct effects of the different TKIs (Figure 2, dotted lines). The most potent aggregation inhibition was observed with dasatinib (>90% inhibition with 1 µM, Figure 2A). Surprisingly, the combined SFK and Syk inhibitor fostamatinib, which has several targets in the platelets, did not show a relevant inhibitory effect (less than 20% at a high dose of 100 μM, Figure 2B). For the weaker SFK inhibitors sunitinib and pazopanib, the aggregation in washed platelets was nearly abolished with 33 µM (>90% inhibition, Figure 2C,D) and cabozantinib inhibited this response by 60% at the same dose (Figure 2F). Unexpectedly, vatalanib, which does not have assumed targets in platelets, also inhibited this response by 80% at 33 μM (Figure 2H). The IC_50_ values of these four TKIs were between 6 and 13 µM, and a concentration of 10 µM was used for further experiments (Table S1). Axitinib and lapatinib, which have limited or no TK targets, slightly affected the aggregation response to collagen at the highest dose tested of 100 µM (Figure 2E,G).

As most TKIs are protein-bound in the plasma, dose–response curves were also determined in platelet-rich plasma (PRP; Figure 2, solid lines). Upon collagen stimulation, the strongest inhibitory effect in PRP was also observed with dasatinib (IC_50_ of 0.4 µM and >90% inhibition at 3.3 µM, Figure 2A). In patient studies, plasma concentrations of up to 0.1 µM of dasatinib have been reported [43] and thus this concentration was used in the subsequent experiments (Table S1). Inhibition with fostamatinib also resulted in a small but significant decrease in the aggregation response (±20% inhibition, Figure 2B). For sunitinib, significant inhibition (60%) was reached at 33 µM, which was higher than the dose required in the washed platelets (Figure 2C). The IC_50_ value of 33 µM of sunitinib was used for further analysis in whole-blood experiments. In contrast to washed platelets, the aggregation responses in the presence of pazopanib, cabozantinib, or vatalanib were not inhibited or barely inhibited in PRP (Figure 2D,F,H). In agreement with the responses in washed platelets, axitinib and lapatinib did not alter the collagen-induced aggregation in PRP (Figure 2E,F). For the latter set of compounds, no more than 20% inhibition was observed in PRP; therefore, the highest concentration of 33 µM was used for experiments containing plasma. On the basis of these results and the IC_50_ values, we determined the concentration of each TKI for consecutive assays performed with isolated platelets or whole blood (Table S1).

### 2.3. Whole-Blood Thrombus Formation over (Non-)Collagen Surfaces under Flow Is Most Strongly Affected by High-Affinity TKIs

**Figure 3:**
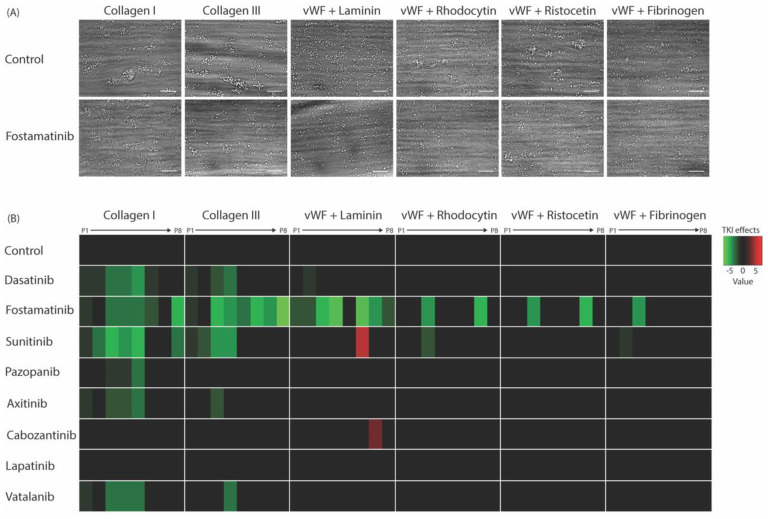
Effects of eight TKIs on whole-blood thrombus formation under flow on different thrombogenic surfaces. Blood samples were pre-incubated with the vehicle or TKI for 10 min. Samples were then recalcified in the presence of PPACK and perfused for 3.5 min at a wall shear rate of 1000 s^−1^ over six different coated surfaces. After perfusion, the formed thrombi were stained with AF568-annexin A5, FITC-α-fibrinogen, and AF647-α-CD62P to detect PS exposure, integrin αIIbβ3 activation, and P-selectin expression, respectively. (**A**) Representative brightfield images of control or fostamatinib-treated blood after flow over collagen Type I, collagen Type III, vWF plus laminin, vWF plus rhodocytin, vWF plus ristocetin, or vWF plus fibrinogen. (**B**) Subtraction heatmap representing significantly altered effects of parameters P1–P8 (normalized and analyzed as in Figure S2). Note: P1, morphological score of platelet adhesion and thrombus formation; P2, surface area coverage of the adhered platelets; P3, platelet aggregate contraction score; P4, platelet aggregate multilayer score; P5, coverage of multi-layered platelet aggregation; P6, αIIbβ3 activation; P7, P-selectin expression; and P8, PS exposure. The results from the vehicle control runs were set at 0. Effects were filtered for significant alterations (*p* < 0.05) induced by TKIs.

To explore the effects of the TKIs under physiologically relevant circumstances, we investigated platelet activation and thrombus formation under flow on coated surfaces, triggering a range of platelet receptors, i.e., GPVI, GPIb, CLEC-2, and the integrins α6β1 and αIIbβ3. Blood samples were pre-incubated with one of the TKIs (mostly applied at 33 μM; see Table S1) and perfused over multiple microspots containing collagen Type I or III, or vWF co-coated with either laminin, rhodocytin, ristocetin, or fibrinogen. Microscopic analysis of brightfield and fluorescence images resulted in five different thrombus parameters (P1–5) and three platelet activation markers (P6–8; Figure S2). In agreement with previous results [44], different types of platelet thrombi were formed on various surfaces under the control condition (Figure 3A). A systematic analysis was performed for evaluating the effects of each TKI by generating a subtraction heatmap for the eight different parameters, which were scaled (0–10) for each parameter and each surface (Figure 3B). In contrast to the limited effect on collagen-induced aggregation, fostamatinib at 33 µM was most potent in suppressing thrombus formation and platelet activation under flow on collagen as well as non-collagen surfaces by approximately 50% (Figure 3A,B). Dasatinib at 0.1 µM reduced collagen-induced thrombus height and contraction under flow by approximately 50%, while platelet deposition and activation were not significantly affected. On the vWF co-coated surfaces, this concentration of dasatinib did not affect thrombus parameters (Figure 3B). Sunitinib, in particular, affected multiple parameters of thrombus formation on the two collagen surfaces by 10–50% but showed limited effects on other surfaces. Pazopanib, axitinib, and vatalanib showed weak inhibition (10–20%), only affecting one or two parameters on collagen Type I and III. Mainly, the size, height, and density of the thrombi were reduced (Figure 3B and Figure S2). The inhibition of thrombus formation by dasatinib or sunitinib was in line with the effects seen in the aggregation of PRP. Moreover, the weaker effects of the other TKIs (pazopanib, axitinib, cabozantinib, lapatinib, and vatalanib; Figure 3B) were in agreement with the PRP aggregation results. Together, these results indicated that the TKIs with the highest affinities for kinase targets in platelets (dasatinib, fostamatinib, and sunitinib) had the greatest impact on whole-blood thrombus formation under flow.

### 2.4. Differential Effects of TKIs on GPVI-Induced Calcium-Signaling

**Figure 4:**
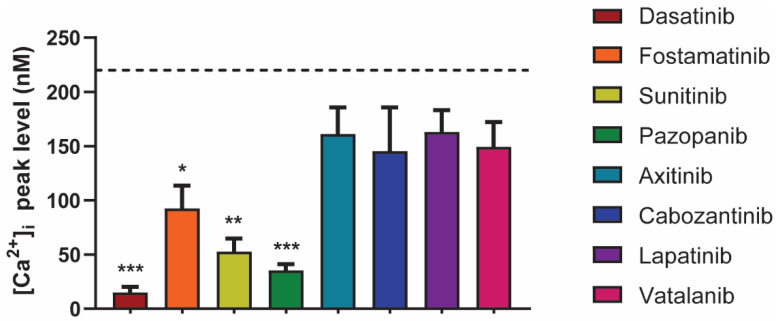
Effects of different TKIs on GPVI-induced intracellular calcium responses. Fura-2-loaded platelets (200 × 10^9^/L) were pre-incubated with vehicle, dasatinib (100 nM), fostamatinib (33 μM), sunitinib (10 μM), pazopanib (10 μM), axitinib (33 μM), cabozantinib (10 μM), lapatinib (33 μM), or vatalanib (10 μM) for 10 min and stimulated with CRP-XL (10 µg/mL) in the presence of 2 mM CaCl_2_. The histograms show the maximal rises in [Ca^2+^]_i_ (*n* = 6). The black dotted line indicates the average of the control values. Data are shown as means ± SEM; * *p* < 0.05, ** *p* < 0.01, and *** *p* < 0.001.

To further explore the inhibitory effects of the TKIs on platelet-signaling, we measured the GPVI-induced intracellular calcium responses. Platelets were loaded with the probe Fura-2, pre-incubated with the vehicle or TKI, and then stimulated by the GPVI agonist CRP-XL. Notably, dasatinib, fostamatinib, sunitinib, and pazopanib significantly decreased or even abolished the maximum intracellular calcium signal by >90%, 57%, 77%, and 82%, respectively (Figure 4). In contrast, intracellular calcium rises were unchanged in the presence of axitinib, cabozantinib, lapatinib, and vatalanib (Figure 4). Similar effects were seen when comparing the slope and area under the curve of these responses (Figure S3). In general, these results are in line with the observed effects on collagen-induced aggregation. However, cabozantinib and vatalanib showed 30–70% inhibition of platelet aggregation. This suggested that for dasatinib, fostamatinib, sunitinib, and pazopanib, the effects were mediated by early GPVI-induced signaling events in accordance with Syk or SFK inhibition, whereas for cabozantinib and vatalanib, the anti-aggregatory effects, were downstream of the calcium rises.

### 2.5. Diverse Effects of Fostamatinib, Cabozantinib, and Vatalanib on Platelet Activation Markers and Platelet Populations

**Figure 5:**
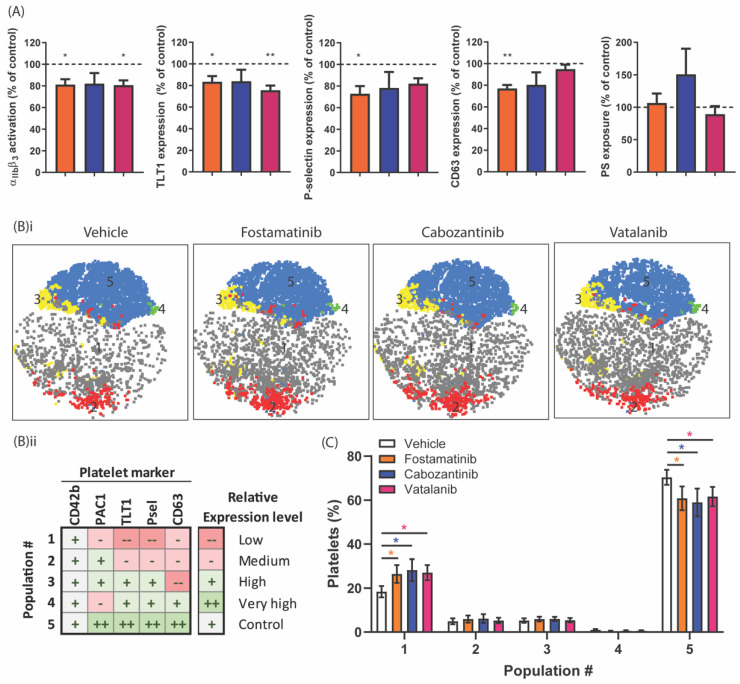
Effect of TKIs on populations of activated platelets in response to GPVI stimulation. Washed platelets (25 × 10^9^/L) were incubated with vehicle, fostamatinib (33 µM), cabozantinib (33 µM), or vatalanib (33 µM) for 10 min. Platelet activation was induced by 5 µg/mL of CRP-XL for 15 min and simultaneously labeled for activated integrin αIIbβ3 (PAC1-FITC), α-granule secretion (αTLT1-BV421 and αCD62P-PerCP Cy5.5) and δ-granule/lysosome secretion (αCD63-APC). Separately, PS exposure was induced using 5 µg/mL of CRP-XL, in addition to 4 nM of thrombin for 60 min at 37 °C, labeled by annexin A5-PE. (**A**) Histograms of integrin α_IIb_β_3_ activation, TLT1, P-selectin, and CD63 expression, as well as PS exposure, are shown as percentages of the control condition. (**B**) Distribution profiles of the platelet populations after stimulation by CRP-XL. (**i**) Two-dimensional plots visualizing the platelet fractions per population in the presence of vehicle, fostamatinib, cabozantinib, or vatalanib. (**ii**) Characterization of CRP-XL-induced platelet populations based on the relative expression levels of activation markers determined by FlowJo analysis using the tSNE and FlowSOM plugins (see Table S2). (**C**) Histograms visualizing the abundance of a certain platelet population (FlowSOM) found in a particular platelet fraction (conditions: vehicle (white bars) or fostamatinib, cabozantinib, or vatalanib (colored bars)). The sum of all the population abundance percentages of a fraction (e.g., the vehicle) amounts to 100% platelets. Data are shown as means ± SEM (*n* = 6); * *p* < 0.05 and ** *p* < 0.01.

In order to further explain the selective anti-aggregatory effects of fostamatinib, cabozantinib, and vatalanib, we investigated their effects on GPVI-induced integrin αIIbβ3 activation, as well as on the secretion of α and δ-granules and lysosomes. In general, all platelet activation markers were reduced by about 20% in the presence of cabozantinib, vatalanib, and fostamatinib (at 33 µM, Figure 5A). Fostamatinib consistently inhibited all GPVI-induced activation responses by 20–25%. In contrast, PS exposure was not inhibited by fostamatinib in isolated platelets (Figure 5A). The effects of cabozantinib and vatalanib on CRP-induced platelet activation were more limited. Both cabozantinib and vatalanib inhibited integrin αIIbβ3 activation by 20%, in line with the inhibition of collagen-induced aggregation in washed platelets. Furthermore, cabozantinib inhibited δ-granule and lysosome secretion, whereas vatalanib resulted in a reduction in α-granule secretion. PS exposure was also not affected by cabozantinib and vatalanib. As sustained high calcium rises are a prerequisite for PS exposure, the absence of any effects on PS exposure by the TKIs agrees with the calcium responses, which remained unchanged by cabozantinib and vatalanib. Hence, cabozantinib and vatalanib suppress platelet integrin activation and secretion at doses that do not influence the calcium response. 

To evaluate the differential sensitivity of platelets towards these TKIs in terms of activation markers, we determined how the formation of activated platelet populations was influenced by the presence of fostamatinib, cabozantinib, or vatalanib. Comparison of the four different activation markers, reflecting the different stages of granular secretion and integrin activation, produced up to five different populations upon stimulation of GPVI. The changes in the distribution of these populations in the presence of the TKIs are illustrated by 2D scatterplots that depict the distribution of the populations (Figure 5B(i)). These ranged from low to very high expression levels for all markers, with equal expressions of the control marker GPIb in all the samples (Figure 5B(ii), Table S2). The abundance of platelets in Population 1, which had low-level platelet activation based on the expression levels of the markers, appeared to be significantly increased by approximately 39% in the presence of fostamatinib, cabozantinib, or vatalanib upon GPVI stimulation (Figure 5C). Conversely, in Population 5, which had high activation of αIIbβ3 and high secretion of α, δ-granules and lysosomes decreased (15%) in the presence of these TKIs (Figure 5C). This approach demonstrated the simultaneous inhibition of secretory and integrin-activating platelet responses by these TKIs. 

**Figure 6:**
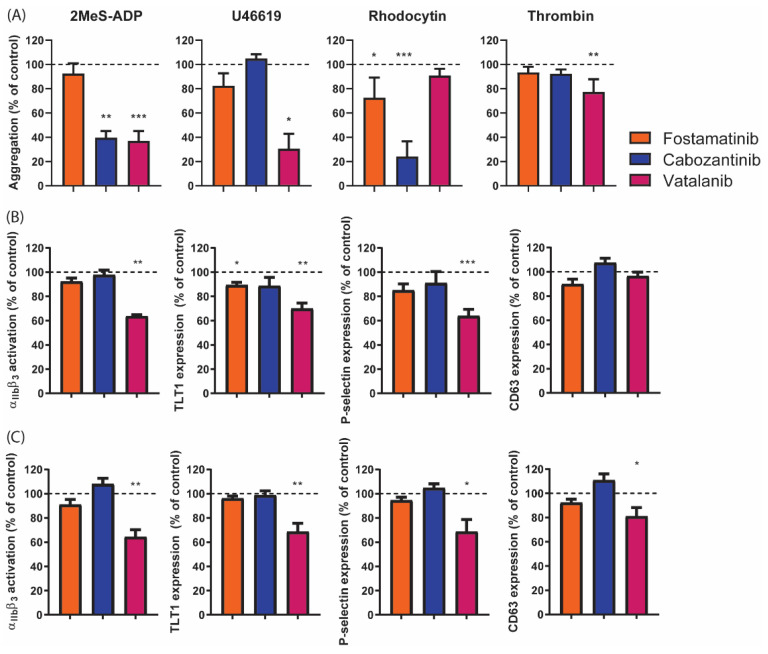
Platelet aggregation and activation induced by different activation pathways in the presence of different TKIs. (**A**) Washed platelets (250 × 10^9^/L) were incubated with vehicle (control), fostamatinib (33 µM), cabozantinib (33 µM), or vatalanib (33 µM) for 10 min. Platelet aggregation was then induced with 1 µM of 2MeS-ADP, 1 µM of U46619 (thromboxane A2 analogue), 1 µg/mL of rhodocytin, or 4 nM of thrombin. Aggregation responses were measured for 8 min. Histograms show aggregation as a percentage of the control (dotted line). (**B**,**C**) Washed platelets (25 × 10^9^/L) were incubated with the vehicle (control) or TKI for 10 min. Platelets were activated by (**B**) 2 µM of 2MeS-ADP or (**C**) 4 nM of thrombin for 15 min. Simultaneous labeling was performed for activated integrin αIIbβ3 (PAC1-FITC), α-granule secretion (αTLT1-BV421, αCD62P-PerCP Cy5.5), and δ-granule/lysosome secretion (αCD63-APC). Histograms for integrin αIIbβ3 activation, TLT1, P-selectin, and CD63 expression are given as percentages of the control condition (dotted line). Data are shown as means ± SEM (*n* = 6); * *p* < 0.05, ** *p* < 0.01, and *** *p* < 0.001.

In whole-blood perfusion experiments, we observed inhibitory effects of fostamatinib on thrombus formation on vWF co-coated surfaces. To determine the effects of fostamatinib, cabozantinib, and vatalanib on platelet activation induced by other agonists, platelet aggregation was investigated in response to ADP, the thromboxane A2 analog U46619, or the CLEC-2 agonist rhodocytin or thrombin. Fostamatinib only reduced rhodocytin-induced aggregation by 25% (Figure 6A), in agreement with Syk-signaling under CLEC-2. In contrast, cabozantinib significantly inhibited the aggregation to ADP (60%) and rhodocytin (75%), while vatalanib prominently reduced the responses to ADP (60%) and U46619 (70%), and slightly reduced the response to thrombin (20%, Figure 6A). In line with these aggregation results, vatalanib suppressed ADP and thrombin-induced integrin αIIbβ3 activation, TLT1 expression, and P-selectin expression by 30–40% (Figure 6B,C). Upon thrombin stimulation, the δ-granule and lysosome secretion were also partly inhibited (Figure 6C). In contrast to the observed reduction in the ADP-induced aggregation with cabozantinib, no inhibition of platelet activation was observed in response to ADP (Figure 6B,C). Taken together, these results suggest that the latter two compounds inhibited signaling entities in pathways other than that induced by GPVI.

## 3. Discussion

In this work, we explored the antiplatelet properties of the TKIs dasatinib, fostamatinib, sunitinib, pazopanib, cabozantinib, axitinib, lapatinib, and vatalanib to investigate the possible repurposing of these compounds as antiplatelet drugs. We observed that the TKIs with the highest reported affinities for platelet-expressed molecular targets (dasatinib, fostamatinib, and sunitinib) most strongly inhibited platelet function. Dasatinib, fostamatinib, sunitinib, and pazopanib interfered in early collagen receptor-induced molecular-signaling compared to cabozantinib and vatalanib. 

Overall, in this study, we observed that the TKIs with the strongest affinities for targets in platelets at the optimal inhibitory dose showed the greatest inhibition of collagen-dependent whole-blood thrombus formation under flow. Furthermore, fostamatinib was the most effective inhibitor of thrombus formation on vWF plus rhodocytin or vWF plus laminin. In the presence of other TKIs, thrombus formation was no more than mildly reduced and only on collagen. Intracellular calcium measurements and activation responses in isolated platelets showed that the inhibitory effects of dasatinib, fostamatinib, sunitinib, and pazopanib were in line with the proposed inhibitory effects on Syk and/or SFK, which are downstream of GPVI-induced platelet activation. Calcium measurements indicated that the effects of the low-affinity inhibitors cabozantinib and vatalanib were downstream of phospholipase Cγ phosphorylation. 

The strong effects seen in the present study of the active metabolite of fostamatinib (R406), which has high affinity for Syk on thrombus formation and PS exposure under flow, are in line with a previous study using the selective Syk inhibitor PRT-060318 [45]. However, our current results obtained in isolated platelets are in contrast to former studies that demonstrated a significant inhibition of collagen or rhodocytin-induced platelet aggregation by low concentrations of R406 [26,46]. In this study, we observed only minor inhibition at higher concentrations. The difference may be due to the longer incubation time of the platelets with the inhibitor in the present study. As fostamatinib is a reversible inhibitor, the incubation time could be of importance; however, we observed strong inhibition in whole-blood perfusion experiments using the same incubation time. Furthermore, other studies have reported incubation with fostamatinib for even longer time periods in platelets as well as in other cell types, making transient inhibition unlikely [27,47]. In addition, here, we observed that fostamatinib strongly inhibited GPVI-induced PS exposure in whole-blood thrombus formation under flow, whereas in isolated platelets, this response was not affected. PS exposure is dependent on sustained high intracellular calcium levels [48], which we showed to be inhibited by fostamatinib. As PS exposure was triggered by dual stimulation of CRP and thrombin in the present setting, this might have overcome the partly decreased calcium signal induced by GPVI stimulation in the presence of fostamatinib, thus resulting in normal PS exposure. 

Several of the investigated TKIs are used for treating metastatic renal cell carcinoma (mRCC) patients, i.e., sunitinib, pazopanib, cabozantinib, and axitinib. The current results with sunitinib and pazopanib are consistent with a moderate inhibition of SFK, Syk, and other relevant tyrosine kinases in platelets (Figure 1). For both compounds, only mild bleeding effects have been reported [31]. Cabozantinib is used mainly as a second or third-line treatment for mRCC patients, while axitinib (combined with pembrolizumab) is used as a first-line mRCC treatment, but their effects on platelets have not been investigated thus far. For cabozantinib, here, we reported a reduced collagen, CLEC-2, and ADP-induced aggregation in washed platelets, but no effects on thrombus formation. This is in line with the only limited bleeding reported in another study [31]. Axitinib, used here at the dose of 33 µM, was essentially ineffective in most assays. We observed limited effects on thrombus height and contraction in whole blood perfused over collagen. Notably, axitinib was previously described to induce mild (and, in rare cases, severe) thrombocytopenia in 15–20% of patients [49,50,51], which points to a megakaryocytic defect rather than a platelet function defect contributing to the reported bleeding [52,53]. 

Lapatinib and vatalanib have been formerly reported to have limited affinity for tyrosine kinases in platelets and no bleeding has been reported [24]. In agreement with the study by Li et al. [54], lapatinib did not alter thrombus formation in whole-blood perfusion, as well as intracellular calcium responses and aggregation in isolated platelets in the present study. Surprisingly, in the presence of vatalanib, here, we observed an inhibition of the thrombus size, height, and contraction on collagen under flow at 33 µM, which is in contrast to the observations reported in the study of Li et al., but the concentration used was not reported in their study [54]. In addition, we detected that in isolated platelets, vatalanib inhibited collagen, ADP, TxA_2_, and thrombin-induced aggregation, as well as platelet activation. As GPVI-induced intracellular calcium rises were not affected by vatalanib, this suggested that vatalanib affects platelet-signaling downstream from calcium. Vatalanib was developed to inhibit all VEGF receptors and is mostly used for treating patients with colorectal cancer [55]. As no platelet tyrosine kinases have been reported to be affected by this compound, further research is required to investigate the inhibitory mechanism in platelets.

The dose–response curves showed the distinct effects of the different TKIs both in isolated platelets and in the presence of plasma. In washed platelets, dasatinib, sunitinib, pazopanib, cabozantinib, and vatalanib prominently inhibited collagen-induced aggregation, while in PRP, this response was unaffected (cabozantinib and pazopanib) or required a higher concentration of the TKI to achieve inhibition (dasatinib, sunitinib, and vatalanib). For pazopanib, sunitinib, and dasatinib, this is in line with previously reported findings [28,30,56] and can be explained by high plasma-binding [34,57,58,59]. Indeed, in patients undergoing TKI treatment, the bioavailability of the drug is considered to be influenced by plasma binding, with implications for drug toxicity and response [60]. Based on the dose–response curves, regarding the IC_50_ values and the plasma concentrations in patients, in this study, we mostly used physiologically relevant concentrations for either isolated platelets or platelets in the presence of plasma (Table S1). In line with this, for the TKIs that did not inhibit or only partly inhibited collagen-induced aggregation in PRP, we also observed no or only limited effects on thrombus formation under flow.

For the eight TKIs investigated in the present report, we observed different effects throughout the diverse platelet function assays. We speculate that these differences may be due to a combination of several possible differences among the TKIs. Firstly, the affinity profile of each TKI for the relevant tyrosine kinases implicated in platelet activation (Figure 1) will determine the effectiveness of the TKI in each assay. This affinity profile may also be related to the molecular structure (Figure S1) and classification of the TKI with regard to its mode of action [24]. The TKIs investigated in the present study all belong to Type I, except for cabozantinib (Type II) and vatalanib (Type III). With regard to the effects on intracellular calcium rises, all TKIs that showed a significant reduction belong to Type I, but so do axitinib and lapatinib, which did not affect this response (Figure 4). Therefore, a structure–function relationship cannot be deduced from these measurements. Finally, as discussed above, the presence of plasma also influenced the effect of a TKI in a specific assay.

For fostamatinib, cabozantinib, and vatalanib, we investigated the effects on secretion and integrin activation in more detail. The most consistent effects were observed with fostamatinib, which moderately inhibited all GPVI-induced activation responses. Cabozantinib and vatalanib inhibited integrin αIIbβ3 activation, along with aggregation. Interestingly, cabozantinib mildly suppressed δ-granule and lysosome secretion, whereas vatalanib reduced α-granule secretion. The reason for this difference is unclear. Furthermore, we used two markers to measure α-granule secretion, namely P-selectin (CD62P) and TLT1. Although, in general, we observed similar results using both markers with several agonists as well as inhibitors, it has been reported by others that TLT1 is a more sensitive marker than P-selectin for platelet activation [61]. When soluble TLT1 is released in the plasma, it has been described as enhancing platelet aggregation [62,63] and has been demonstrated to regulate early clot formation through the stabilization of αIIbβ3 outside–in-signaling [64]. Hence, this may provide an additional mechanism of the inhibitory effect of vatalanib.

We also determined the distribution of the individual platelet activation markers in different populations to further focus on the effects of fostamatinib, cabozantinib, or vatalanib on GPVI-induced platelet activation. In general, in the presence of TKIs, the populations with highly expressed activation markers decreased, whereas the populations with negative expressions of these markers increased. This integrated approach suggested that pre-incubation with TKIs may affect the general responsiveness of the previously described heterogeneous platelet populations [65].

A limitation of the present study is that all TKIs were added in vitro to isolated platelets or whole blood obtained from healthy donors. It would be very relevant to directly observe the effects of these TKIs on platelets in blood obtained from cancer patients undergoing treatment compared with that of untreated cancer patients. Furthermore, we did not investigate the direct effects of the TKIs on the phoshorylation of platelet tyrosine kinases after stimulation with different agonists, e.g., by western blotting in combination with the 4G10 antibody or by the PamGene kinase assay. This latter assay has successfully shown all tyrosine kinases that were affected by sunitinib in platelets stimulated by CRP [30]. A direct comparison of the effects of all TKIs on all platelet tyrosine kinases would provide new insights into the affected platelet targets, as well as into possible structure–function relationships, which could further explain the results of the present study.

The aim of the present study was to assess the potential for repurposing TKIs as antiplatelet drugs, which would be a direct potential application of the results. This is important because there is a need for novel antiplatelet drugs that inhibit thrombosis but do not interfere in hemostasis [5]. TKIs are orally available compounds that have been shown to be well tolerated and, in general, cause only a mild increase in bleeding risk. Hence, for the repurposing of TKIs as antiplatelet drugs, these compounds first should be tested at different doses in cardiovascular patients. As many TKIs have been developed for cancer treatment [24,25], future studies should not be limited to the eight TKIs investigated here but should include all TKIs with a minor reported bleeding risk. 

In conclusion, in this study, we observed that fostamatinib was the most effective inhibitor of thrombus formation on collagen under flow and that pazopanib, sunitinib, dasatinib, axitinib, and vatalanib also mildly reduced this process. Furthermore, the TKIs showed variable inhibiting effects in isolated platelets stimulated by several agonists. As fostamatinib, sunitinib, pazopanib, and vatalanib, in particular, have been associated with mostly manageable or no bleeding events, these may be promising candidates to explore further as antiplatelet drugs. Clinicians should be aware of the antiplatelet properties of TKIs in general, especially when patients are already being treated with antiplatelet or anticoagulant drugs.

## 4. Materials and Methods

### 4.1. Materials

The active metabolite of fostamatinib (R406) was obtained from InvivoGen (San Diego, CA, USA). Active metabolites of axitinib (A-1107), cabozantinib, dasatinib, and pazopanib were purchased from LC Laboratories (Woburn, MA, USA). The active metabolite of lapatinib (GW-572016) was purchased from Selleckchem (Houston, TX, USA), whereas the active metabolite of sunitinib was obtained from Pfizer (New York, NY, USA). The active metabolite of vatalanib (PTK787) was obtained from Adooq Bioscience (Irvine, CA, USA). Bovine serum albumin (BSA), D(+)-glucose, unfractionated heparin, and apyrase were purchased from Sigma-Aldrich (Saint Louis, MO, USA). Horm collagen Type I was obtained from Takeda (Hoofddorp, the Netherlands), whereas the agonists collagen-related peptide-crosslinked (CRP-XL) and von Willebrand Factor III (vWF-III) were obtained from CambCol Laboratories (Cambridge, UK). Rhodocytin was purified from *Calloselasma rhodostoma* venom as described previously [66]. Thrombin was obtained from Enzyme Research Laboratories Inc. (South Bend, IN, USA). U46619 (a thromboxane A2 receptor agonist) was obtained from Cayman Chemicals (Ann Arbor, MI, USA). 2-Methylthio-adenosine-diphosphate (2MeS-ADP) and D-phenylalanyl-prolyl-arginyl chloromethyl ketone (PPACK) were obtained from Santa Cruz Biotechnology (Dallas, TX, USA). Laminin was purchased from Octapharma (Lachen, Switzerland), whereas Fura-2-AM and allophycocyanin (APC)-labeled mouse anti-human CD63 monoclonal antibody (mAb, clone MEM-259) were obtained from Invitrogen, Fisher Scientific (Carlsbad, CA, USA). Fluorescein isothiocyanate (FITC)-labeled PAC1 mAb against activated human integrin αIIbβ3 (340507), phycoerythrin (PE)-labeled annexin A5, Brilliant Violet (BV)510-conjugated mouse anti-human CD42b mAb (clone HIP1), and BV421-labeled rat anti-human TLT-1 mAb (clone 268420) were purchased from BD Bioscience (Franklin Lakes, NJ, USA). FITC-conjugated α-fibrinogen mAb was purchased from DAKO (F0111; Santa Clara, CA, USA). Alexa Fluor (AF)568-conjugated annexin A5 was purchased from Molecular Probes, Life Technologies (New York, NY, USA). Peridinin–chlorophyll–protein cyanine 5.5 (PerCP Cy5.5)-conjugated mouse anti-human CD62-P mAb (clone AK4) and AF647-labeled mouse anti-human CD62-P mAb (clone AK4) were obtained from Biolegend (London, UK). FITC-conjugated annexin A5 was obtained from Pharmatarget (Maastricht, the Netherlands). 

### 4.2. Blood Collection and Platelet Isolation

In accordance with the Declaration of Helsinki and approval by the local medical ethical committee (Maastricht University Medical Centre+, MUMC+), we obtained full informed consent from all participants. Blood from healthy volunteers was collected in 3.2% trisodium citrate tubes by venipuncture after discarding the first 3 mL of blood. Blood cell counts and hematological parameters were assessed using a Sysmex XP300 (Kobe, Japan). 

Platelet-rich plasma (PRP) or platelets were isolated from whole blood as described previously [67,68]. Washed platelets were resuspended in a Hepes buffer, pH 7.45 (10 mM Hepes, 136 mM NaCl, 2.7 mM KCl, 2 mM MgCl_2_, 1 mg/mL glucose, and 1 mg/mL bovine serum albumin). Platelet count was adjusted as stated per assay.

### 4.3. Thrombus Formation

Thrombus formation under flow was determined as described [44]. Briefly, glass coverslips were coated with 3 microspots of collagen Type I (100 µg/mL), collagen Type III (100 µg/mL), vWF (50 µg/mL) co-coated with laminin (100 µg/mL), vWF co-coated with rhodocytin (250 µg/mL), vWF co-coated with ristocetin (250 µg/mL), or vWF co-coated with fibrinogen (250 µg/mL), and were mounted in a parallel plate flow chamber. Citrated blood samples were incubated with the TKIs axitinib, cabozantinib, dasatinib, fostamatinib, lapatinib, pazopanib, sunitinib, or vatalanib, or the vehicle for 10 min at room temperature. Pre-incubated whole-blood samples were recalcified in the presence of PPACK (40 µM) and perfused at a wall shear rate of 1000 s^−1^ for 3.5 min. Platelet activation properties were determined by post-perfusion with the Hepes buffer (supplemented with 2 mM of CaCl_2_ and 1 U/mL of heparin) containing FITC-conjugated α-fibrinogen mAb (1:80), AF647-conjugated αCD62P mAb (1:100), and AF568-conjugated annexin A5 (1:200). Brightfield and fluorescence images were captured using an EVOS microscope (Bothel, WA, USA).

### 4.4. Analysis of the Microscopic Images

Microscopic images were analyzed using specific scripts in the open-access Fiji software (Laboratory for Optical and Computational Instrumentation, University of Wisconsin-Madison, WI, USA) as described [44,69]. Multiple thrombus formation parameters were obtained from brightfield images as follows: P1, morphological score of platelet adhesion and thrombus formation (scale: 0–5); P2, surface area coverage of the adhered platelet (% SAC); P3, platelet aggregate contraction score (scale: 0–3); P4, platelet aggregate multilayer score (scale: 0–3); and P5, coverage of multi-layered platelet aggregation (% SAC). From the fluorescence images, platelet activation parameters were obtained by determining: P6, integrin αIIbβ3 activation (% SAC); P7, P-selectin expression (% SAC); and P8, phosphatidylserine (PS) exposure (% SAC). Heatmaps were generated from the mean values for each parameter and each surface for comparative data analysis. Values were scaled from 0 to 10 on the basis of the highest value of each parameter for each surface. For visualization of the effects, subtraction heatmaps were generated by subtraction of the scaled control data from each TKI condition and the heatmaps were filtered on the basis of significant differences (*p* < 0.05). Heatmaps were generated using R (i386 3.2.5; Vienna, Austria).

### 4.5. Platelet Aggregation by Light Transmission Aggregometry

Isolated platelets or PRP (250 × 10^9^ platelets/L) were incubated with TKIs or the vehicle for 10 min at 37 °C. Maximal aggregation was induced by collagen Type I (1 µg/mL), 2MeS-ADP (1 µM), U46619 (1 µM), rhodocytin (1 µg/mL), or thrombin (1 nM). Platelet aggregation was recorded using a Chronolog optical aggregometer (Havertown, PA, USA). The maximum amplitude was quantified at 8 min after the addition of the agonist. 

### 4.6. Cytosolic Ca^2+^ Measurements

Washed platelets (200 × 10^9^ platelets/L) were loaded with Fura-2 acetoxymethyl ester and changes in cytosolic Ca^2+^ ([Ca^2+^]_i_) were measured in 96-well plates using a FlexStation 3 (Molecular Devices, San Jose, CA, USA) as described in [45,70]. Briefly, platelets in the suspension were pre-incubated with TKIs or the vehicle for 10 min at room temperature. Pre-loaded and pre-incubated samples were stimulated by CRP-XL (10 µg/mL). Changes in Fura-2 fluorescence were measured in duplicates and ratio values were calculated. Data are presented as [Ca^2+^]_i_ in nM or as the area under the curve (AUC) in nMs.

### 4.7. Platelet Activation by Flow Cytometry

Washed platelets (25 × 10^9^ platelets/L) were incubated with TKIs or the vehicle for 10 min at room temperature. Platelets were stimulated in the presence of 2 mM of CaCl_2_ by CRP-XL (5 μg/mL), 2MeS-ADP (2 μM), or thrombin (4 nM) for 15 min and simultaneously stained for multiple activation markers using an antibody mix consisting of FITC-conjugated PAC1 (1:20), BV510-conjugated αCD42b (1:50), BV421-conjugated αTLT1 (1:50), APC-conjugated αCD63 (1:20), and PerCP-Cy5.5-conjugated αCD62P (1:500) mAbs. For measuring phosphatidyl-serine (PS) exposure, platelets were activated with a combination of CRP-XL (5 μg/mL) and thrombin (4 nM) for 1 h at 37 °C, and post-labeled with PE-conjugated annexin A5. Samples for activation markers as well as for PS exposure were fixed after stimulation for at least 30 min at 4 °C in the dark using a 0.3% fixation buffer before measuring 10,000 events per sample on a BD FACSCanto II (BD bioscience, Franklin Lakes, NJ, USA).

### 4.8. Analysis of Flow Cytometry Data

Separate FCS files were opened in FlowJo V10 software (Treestar, Ashland, OR, USA) and checked for data anomalies by FlowAI [71]. The event count per sample was set at 5000 events by DownSample for equal weighting of every sample in the final concatenated files. Samples were concatenated for all agonists and for both vehicle and TKI treatments. The resulting files were subjected to t-distributed stochastic neighbor embedding (tSNE) analysis to reduce the high dimensionality to a 2D plot. The concatenated file for the vehicle was subjected to FlowSOM analysis to create 5 clusters; then, the concatenated TKI file was mapped against the FlowSOM results of the vehicle treatment [72] to obtain the same population distribution for the vehicle and TKI conditions. The fractions of platelets present in the resulting FlowSOM populations were determined. The characteristics of the platelet populations were based on their activation markers.

### 4.9. Statistical Analysis

The data are shown as means ± standard error of mean (SEM). GraphPad Prism 8.3.0 software (La Jolla, CA, USA) was used for statistical analysis with an unpaired non-parametric *t*-test (Mann–Whitney) or one-way ANOVA. A *p*-value of less than 0.05 was considered to be statistically significant, where * is *p* < 0.05, ** is *p* < 0.01, and *** is *p* < 0.001.

## Figures and Tables

**Table 1:** Characterization of TKIs used for cancer treatment, with targets present in platelets.

TKI	Reported Targets	Targets in Platelets (MKs)	Types of Cancers Treated	Bleeding Reported	Ref.
Dasatinib	PDGFR, EFGR, BCR-ABL, EphA2, Kit, SFK	SFK: Src, Fyn, Lck, Lyn, Yes, Btk, (Kit)	CP CML, AP MB, LB CML	Yes	[32,33]
Fostamatinib	Syk	Syk, Src, Fgr, Fyn, lck, Lyn, Yes	B-cell lymphoma, CLL	No	[34]
Sunitinib	VEGFR, PDGFR, CSF-R, Ret, Kit, Flt3	(Ret, Kit)	RCC, GIST, PNET	Yes	[29,31,32,35]
Pazopanib	VEGFR, PDGFR, FGFR, Kit, Fms, Itk, Lck	Lck (Kit)	NSCLC, OC, RCC, STS, TC	Yes	[31,32]
Axitinib	VEGFR, PDGFRβ	Lck, Yes, Axl, Tie	RCC	Yes	[31,32]
Cabozantinib	VEGFR, Met, Ret, Kit, Flt3, Axl, Tie	Axl, Tie (Ret, Kit)	TC, RCC	Yes	[31,36]
Lapatinib	EGFR, ErbB1-2	ND	ErbB2^+^ HR^−^ or HR^+^ BC	No	[32,37]
Vatalanib	VEGFR, PDGFRβ, Kit	(Kit)	MCRC	No	[38]

Abbreviations: AP: acute phase; BC: breast cancer; CLL: chronic lymphocytic leukemia; CML: chronic myeloid leukemia; CP: chronic phase; CSF-R: colony-stimulating factor receptor; EGFR: epidermal growth factor receptor; ErbB2: human epithelial growth factor 2; FGFR: fibroblast growth factor receptor; GIST: gastrointestinal stromal tumor; HR: hormone receptor; LB: lymphoid blast; MB: myeloid blast; MCRC: metastatic colorectal cancer; MKs, megakaryocytes; ND: not determined; NSCLC: non-small-cell lung cancer; OC: ovarian cancer; PDGFR: platelet-derived growth factor receptor; PNET: primitive neuroectodermal tumor; RCC: renal cell carcinoma; SFK: Src family kinase; STS: soft tissue sarcoma; TC: thyroid cancer; and VEGFR: vascular endothelial growth factor receptor.

## Data Availability

The data presented in this study are available from the corresponding author upon request.

## Supplementary Materials

Supplementary materials are available online at https://www.mdpi.com/article/10.3390/ijms222011199/s1.
