# Supplementary material for: Multiparameter Evaluation of the Platelet-Inhibitory Effects of Tyrosine Kinase Inhibitors Used for Cancer Treatment
Source: Int J Mol Sci. 2021 Oct 18;22(20):11199. doi: 10.3390/ijms222011199 (PMC8540269; doi:10.3390/ijms222011199)
Supplement: Supplementary file 1 [file ijms-22-11199-s001.zip › ijms-1351684 suppl.pdf]

Multiparameter evaluation of the platelet-inhibitory effects of tyrosine kinase inhibitors used for cancer treatment

Bibian M.E. Tullemans et al.

Supplemental Tables

Table S1. Concentrations of TKIs used in platelet function assays

**TKI**	**Concentration (μM)**	
	**WB or PRP**	**WP**
Dasatinib	0.1	0.1
Fostamatinib	33	33
Sunitinib	33	10
Pazopanib	33	10
Axitinib	75	33
Cabozantinib	33	10 or 33
Lapatinib	33	33
Vatalanib	33	10 or 33

Abbreviations: PRP: platelet-rich plasma; WB: whole blood; WP: washed platelets

Table S2. Cut-off values for platelet markers to determine platelet population characteristics as obtained by FlowSOM.
(**A**) Overview of the median fluorescent intensity (MFI) values given by FlowSOM to characterise each population found, based on the original MFI values, but scaled and transformed by the algorithm.
(**B**) The categories are based on the mean and SD which represent the cut-off values for each marker: "--" values between mean - 1xSD and the lowest value; "-" values between mean and mean - 1xSD; "+" values between mean and mean + 1xSD; "+ +" values between mean + 1xSD and the highest value. (**C**) Transformation of the cut-off values into the categories of levels of expression.

A

		Platelet marker				
		CD42b	PAC1	TLT1	CD62P	CD63
Population #	1	641	264	307	287	301
	2	646	453	404	357	342
	3	636	461	496	483	264
	4	632	284	454	458	394
	5	634	505	569	575	487

B

	Platelet marker				
	CD42b	PAC1	TLT1	CD62P	CD63
Min	-	264	307	287	264
-1xSD	-	282.4	347.5	319.8	270.6
Mean	-	393.4	446.0	432.0	357.6
+1xSD	-	504.4	544.5	544.2	444.6
Max	-	505	569	575	487
SD	-	111.0	98.5	112.2	87.0

C

		Platelet marker				
		CD42b	PAC1	TLT1	CD62P	CD63
Population #	1	+	--	--	--	-
	2	+	+	-	-	-
	3	+	+	+	+	--
	4	+	-	+	+	+
	5	+	++	++	++	++

Legends to supplemental Figures

Figure S1. Molecular structures of tyrosine kinase inhibitors.

Figure S2. Thrombus formation under flow affected by eight TKIs on six different surfaces. Blood samples were pre-incubated with vehicle or TKI for 10 minutes at room temperature. Blood treated with indicated TKI was recalcified in the presence of PPACK, and perfused for 3.5 minutes at a wall shear rate of 1,000 s^{-1} over coated surfaces A-F. Thrombi were post-stained with AF568-annexin A5, FITC-α-fibrinogen and AF647-α-CD62P mAbs to detect PS exposure, integrin activation and P-selectin expression, respectively. Quantification of brightfield and fluorescence images of control and TKI-treated samples with the surfaces (**A**) collagen Type I, (**B**) collagen Type III, (**C**) vWF co-coated with laminin, (**D**) vWF co-coated with rhodocytin, (**E**) vWF co-coated with ristocetin and (**F**) vWF co-coated with fibrinogen. P1, morphological score of platelet adhesion and thrombus formation; P2, surface area coverage of adhered platelets; P3, platelet aggregate contraction score; P4, platelet aggregate multilayer score; P5, coverage of multi-layered platelet aggregation; P6, integrin αIIbβ3 activation; P7, P-selectin expression; P8, PS exposure. The data are shown as means ± SEM (n=6-7). The black dotted lines indicate the average of the control values (<0.1% DMSO); the yellow or green dotted lines indicate the average of the control values (>0.1% DMSO) for sunitinib or pazopanib. * p<0.05.

Figure S3. Effects of different TKIs on GPVI-induced intracellular calcium responses. Fura-2-loaded platelets (200 x 10^9 /L) were pre-incubated with vehicle, dasatinib (100 nM), fostamatinib (33 μM), sunitinib (10 μM), pazopanib (10 μM), axitinib (33 μM), cabozantinib (10 μM), lapatinib (33 μM) or vatalanib (10 μM) for 10 minutes and stimulated with CRP-XL (10 μg/mL) in the presence of 2 mM $CaCl_2$. The histograms show (**A**) the slope of the increase in $[Ca^{2+}]_i$; and (**B**) the area under the curve (AUC), (n=6). The black dotted line indicates the average of the control values. Data are shown as means ± SEM; * p<0.05, **p<0.01, *** p<0.001.
