# Supplementary material for: Multiparameter Evaluation of the Platelet-Inhibitory Effects of Tyrosine Kinase Inhibitors Used for Cancer Treatment
Source: Int J Mol Sci. 2021 Oct 18;22(20):11199. doi: 10.3390/ijms222011199 (PMC8540269; doi:10.3390/ijms222011199)
Supplement: Supplementary file 1 [file ijms-22-11199-s001.zip › ijms-1351684-figures S1-S3.pdf]

Dasatinib

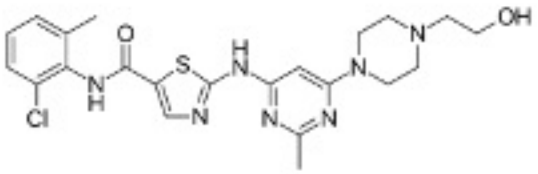

Fostamatinib

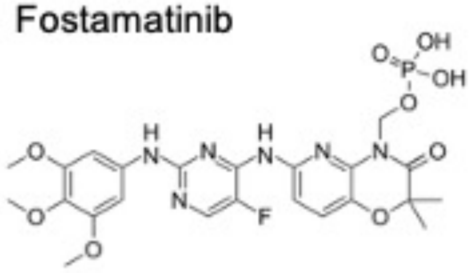

Sunitinib

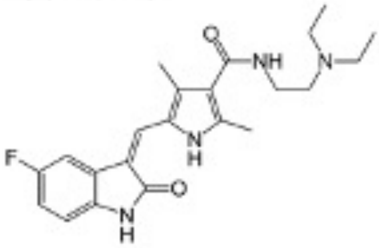

Pazopanib

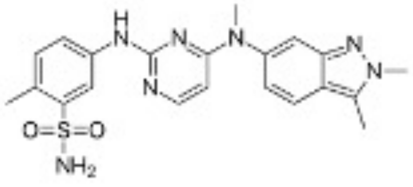

Axitinib

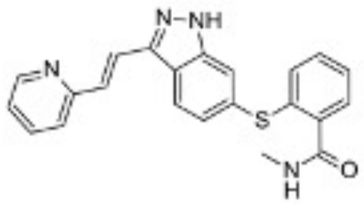

Cabozantinib

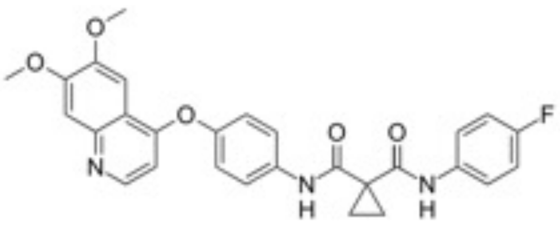

Lapatinib

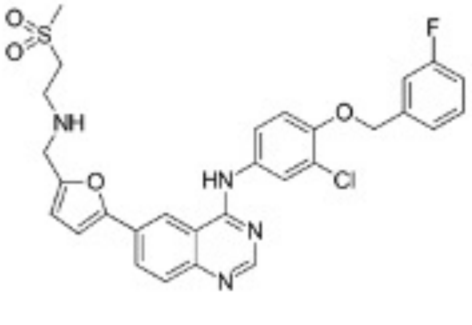

Vatalanib

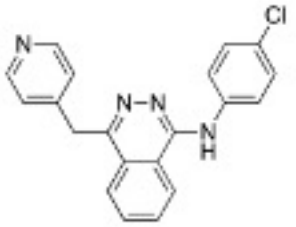

Figure S1. Molecular structures of tyrosine kinase inhibitors.

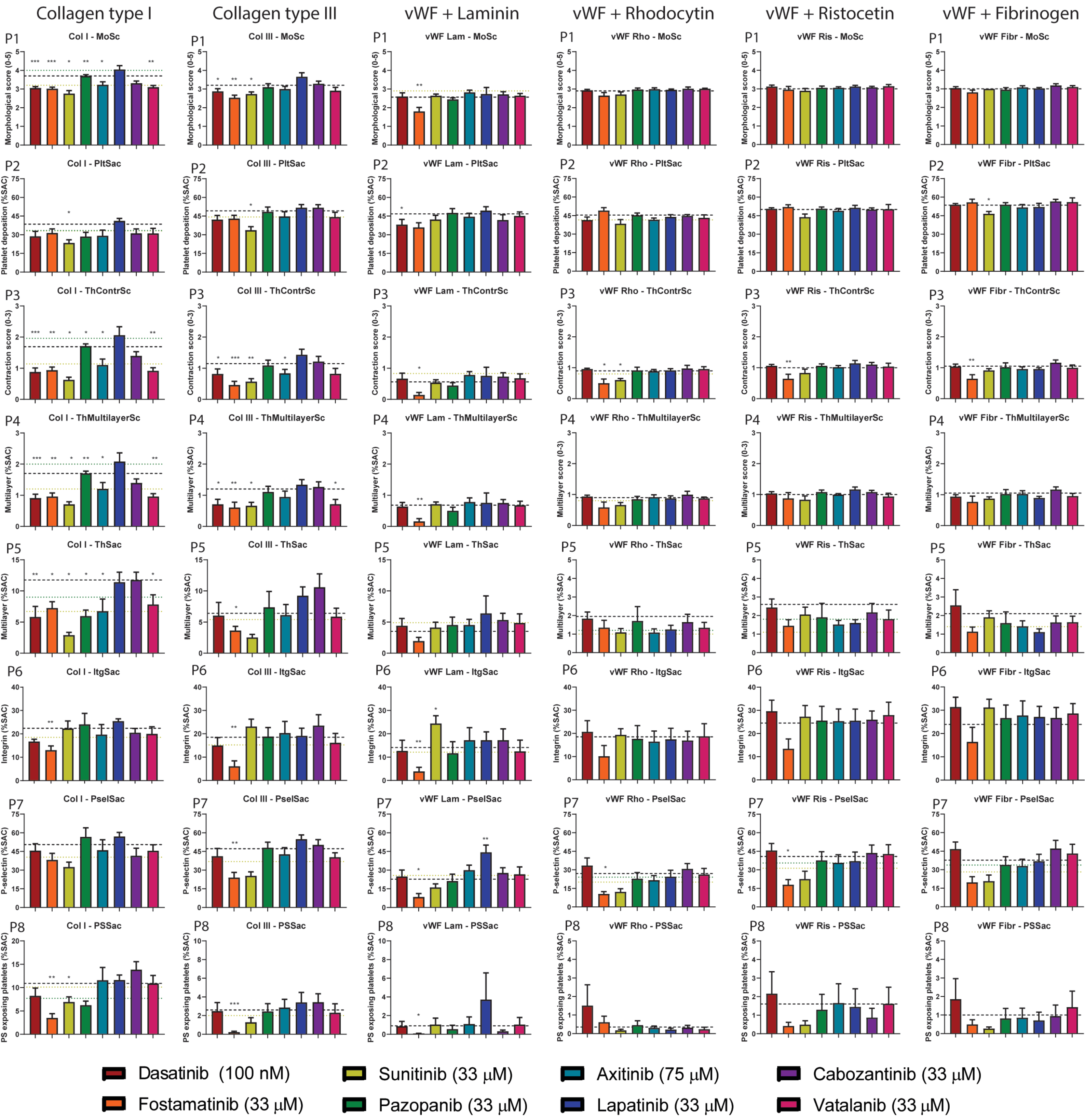
Collagen type I
Collagen type III
vWF + Laminin
vWF + Rhodocytin
vWF + Ristocetin
vWF + Fibrinogen
P1
Col I - MoSc
Col III - MoSc
vWF Lam - MoSc
vWF Rho - MoSc
vWF Ris - MoSc
vWF Fibr - MoSc
Morphological score (0-5)
P2
Col I - PltSac
Col III - PltSac
vWF Lam - PltSac
vWF Rho - PltSac
vWF Ris - PltSac
vWF Fibr - PltSac
Platelet deposition (%SAC)
P3
Col I - ThContrSc
Col III - ThContrSc
vWF Lam - ThContrSc
vWF Rho - ThContrSc
vWF Ris - ThContrSc
vWF Fibr - ThContrSc
Contraction score (0-3)
P4
Col I - ThMultilayerSc
Col III - ThMultilayerSc
vWF Lam - ThMultilayerSc
vWF Rho - ThMultilayerSc
vWF Ris - ThMultilayerSc
vWF Fibr - ThMultilayerSc
Multilayer (%SAC)
Multilayer score (0-3)
P5
Col I - ThSac
Col III - ThSac
vWF Lam - ThSac
vWF Rho - ThSac
vWF Ris - ThSac
vWF Fibr - ThSac
Multilayer (%SAC)
P6
Col I - ItgSac
Col III - ItgSac
vWF Lam - ItgSac
vWF Rho - ItgSac
vWF Ris - ItgSac
vWF Fibr - ItgSac
Integrin (%SAC)
P7
Col I - PselSac
Col III - PselSac
vWF Lam - PselSac
vWF Rho - PselSac
vWF Ris - PselSac
vWF Fibr - PselSac
P-selectin (%SAC)
P8
Col I - PSSac
Col III - PSSac
vWF Lam - PSSac
vWF Rho - PSSac
vWF Ris - PSSac
vWF Fibr - PSSac
PS exposing platelets (%SAC)
Dasatinib (100 nM)
Fostamatinib (33 μM)
Sunitinib (33 μM)
Pazopanib (33 μM)
Axitinib (75 μM)
Lapatinib (33 μM)
Cabozantinib (33 μM)
Vatalanib (33 μM)

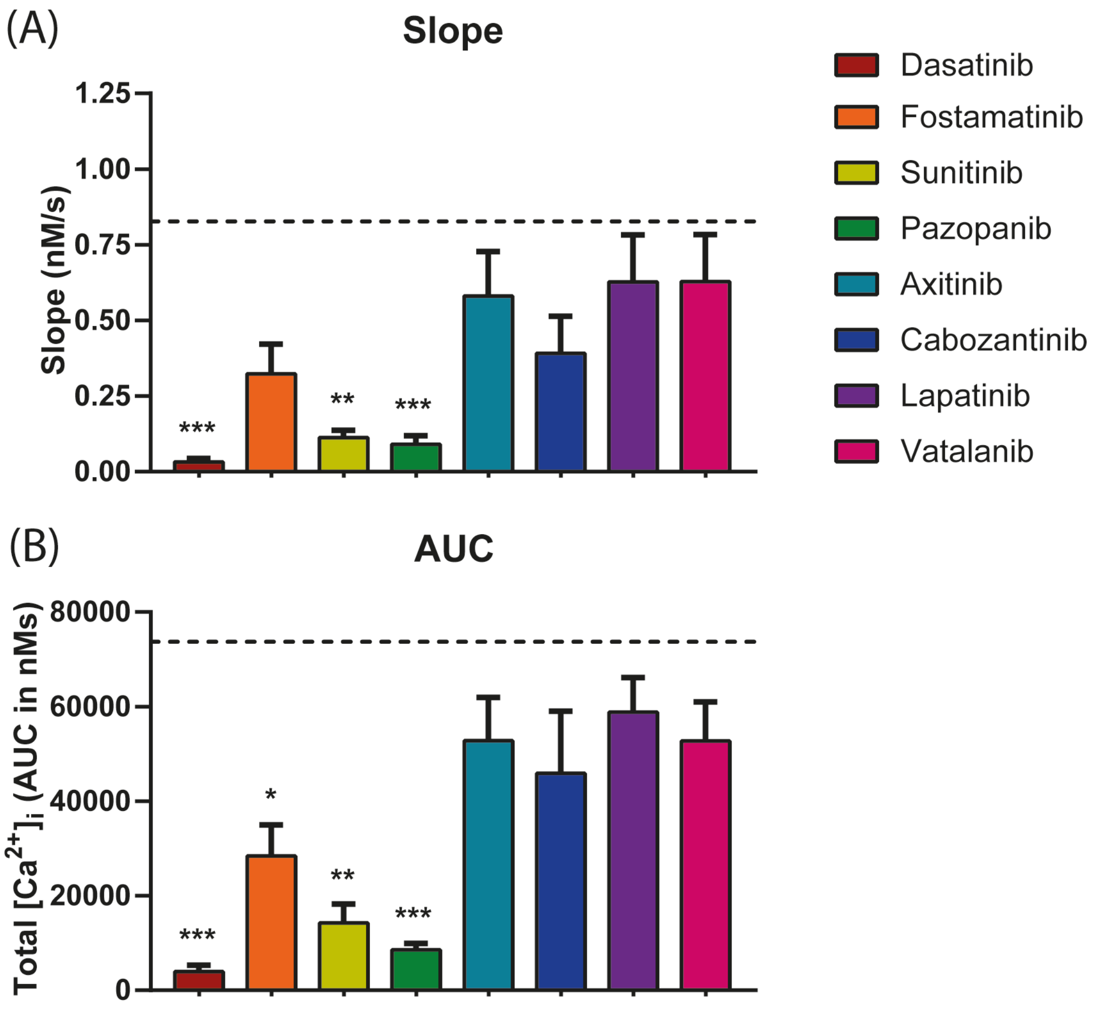

(A)
Slope
Slope (nM/s)
1.25
1.00
0.75
0.50
0.25
0.00
***
**
***
(B)
AUC
Total [Ca2+]i (AUC in nMs)
80000
60000
40000
20000
0
***
*
**
***
Dasatinib
Fostamatinib
Sunitinib
Pazopanib
Axitinib
Cabozantinib
Lapatinib
Vatalanib
